# The association between polypharmacy and mortality in patients with heart failure: Results from the PULSE dataset

**DOI:** 10.1002/ehf2.15445

**Published:** 2025-10-11

**Authors:** Janine Beezer, Andrew L. Clark, Adam Todd, Andrew Kingston, Andrew Husband

**Affiliations:** ^1^ South Tyneside and Sunderland Foundation Trust Sunderland Royal Hospital Sunderland UK; ^2^ School of Pharmacy, The Faculty of Medical Sciences Newcastle University Newcastle upon Tyne UK; ^3^ Hull University Teaching Hospitals NHS Trust, Castle Hill Hospital Cottingham UK; ^4^ School of Pharmacy, The Faculty of Medical Sciences, Newcastle NIHR Patient Safety Collaboration (PSRC) Newcastle University Newcastle upon Tyne UK; ^5^ Population Health Sciences Institute, Faculty of Medical Sciences Newcastle University Newcastle upon Tyne UK

**Keywords:** Heart failure, Polypharmacy, Mortality, Outcomes

## Abstract

**Aims:**

Mortality remains high in heart failure despite advances in heart failure therapy. Heart failure patients are generally older, with multiple long‐term conditions, and polypharmacy is common. This study explores the association between polypharmacy and mortality.

**Methods:**

This retrospective longitudinal observational cohort study collected medication data on admission and discharge from the first heart failure hospitalisation. Association with mortality was explored using Cox proportional hazard models and inverse probability weighting regression analysis.

**Results:**

A total of 660 patients were included, 367 (56%) male, mean age 76.1 (SD ±12.3) and almost 60% (338/660) had died at study end. Median follow‐up time was 2.9 years (25th and 75th quartiles 1.6 and 4.5). It was rare to be discharged from hospital with no polypharmacy (5%, *n* = 31). Heart failure with preserved ejection fraction (HFpEF) was associated with a 32% (HR 1.32, CI 1.08–1.61, *P* = 0.007) higher mortality compared to HFrEF.

In those with heart failure with reduced ejection fraction (HFrEF), univariable analysis showed hyperpolypharmacy was associated with twice the mortality compared to polypharmacy (HR 1.95, CI 1.36–2.82, *P* < 0.001). In multivariable analysis, the association between polypharmacy and mortality was lost. The average treatment effect for hyperpolypharmacy was associated with 26% (Coeff. −0.26, CI −0.43 to −0.09, *P* = 0.003) higher mortality than polypharmacy. The chance of survival to the end of follow‐up was 80% (Coeff. 0.80, CI 0.64–0.95, *P* < 0.01) for those with polypharmacy, and 54% (Coeff. 0.54, CI 0.46–0.61, *P* < 0.01) for those with hyperpolypharmacy.

In HFpEF, hyperpolypharmacy, univariable analysis was not associated with mortality (HR 0.93, CI 0.70–1.24, *P* = 0.63). Average treatment effect also showed that hyperpolypharmacy was not associated with mortality (Coeff. −0.03, CI −0.15 to 0.08, *P* = 0.55). The chance of survival to the end of follow‐up was 67% (Coeff. 0.67, CI 0.58–0.77, *P* < 0.01) with polypharmacy and 64% (Coeff. 0.64, CI 0.57–0.71, *P* < 0.01) with hyperpolypharmacy.

**Conclusions:**

Age, sex, CCI, and CFS are strong mortality predictors for HF irrespective of HF subgroup. Rigorous confounding adjustment suggests polypharmacy is associated with mortality following hospitalisation for HFrEF but not HFpEF. Further studies are needed to address the complex interplay between polypharmacy, age, comorbidity, and frailty.

## Graphical abstract

A study of 660 heart failure patients (mean age 76.1) found that polypharmacy was common, with only 5% discharged without it, and 60% had died by the end of a median 2.9‐year follow‐up. In patients with heart failure with reduced ejection fraction (HFrEF), hyperpolypharmacy was associated with significantly higher mortality, while in those with preserved ejection fraction (HFpEF), it was not. After adjusting for confounders like age, sex, comorbidities, and frailty, polypharmacy remained linked to mortality in HFrEF but not in HFpEF, highlighting the need for further research.

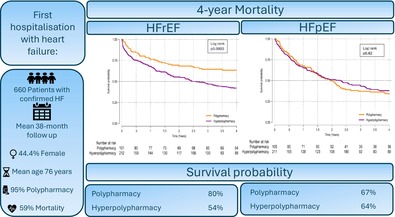

## Introduction

Polypharmacy, traditionally defined as five or more regular medications, is an inevitable consequence of the success of modern medicine. Advances in medical treatment for long‐term conditions mean that terms such as hyperpolypharmacy and super hyperpolypharmacy have been coined for more than 10 medications and more than 15 medications, respectively.^1^ Polypharmacy is associated with increased risk of readmissions and mortality in both patients with heart failure^2^, ^3^ and general populations.^4^, ^5^

Mortality is high in patients with heart failure despite advances in modern heart failure (HF) therapy, with 1‐ and 5‐year survival rates following diagnosis of 76% and 46%, respectively.^6^ Therapeutic interventions for patients with heart failure with reduced ejection fraction (HFrEF) have improved long‐term patient outcomes, which have resulted in changes in guideline‐directed medical therapy (GDMT).^7^, ^8^, ^9^ However, there is less evidence of benefit for the same drugs in patients with heart failure with preserved ejection fraction (HFpEF). The exception is sodium‐glucose cotransporter‐2 inhibitors (SGLT2i): empagliflozin^10^ and dapagliflozin^11^ are associated with a reduction in heart failure hospitalisation. As such, treatment strategies differ between the two pathologies.

Patients with HF are generally older with multiple long‐term conditions (MLTCs). When the conditions are optimally treated, polypharmacy is inevitable, particularly for patients with HFrEF in whom 4‐pillar therapy is indicated. Two systematic reviews have shown an association between polypharmacy and mortality in a general population^12^ and, more recently, in adults over 65 years.^13^ Studies in heart failure have shown similar results following hospital admission.^3^, ^14^ However, such studies do not have such robust criteria for defining heart failure and have not stratified by left ventricular function.

Here, we report the result from the PULSE database, designed to capture medicines data from first heart failure hospitalisation to explore associations with long‐term outcomes.

## Methods

The PULSE (Polypharmacy Use Links Severe Heart Failure Events) dataset has been discussed in detail elsewhere.^15^ It is used to collect data systematically to allow exploration of the association between polypharmacy and mortality following a first admission to hospital with HF.

Data were collected from 660 patients with HF (331 HFrEF and 329 HFpEF). Heart failure admission was defined if the diagnosis in the first coded position using the International Classification of Diseases 10th Revision (ICD‐10) was: I11.0 Hypertensive heart disease with (congestive) heart failure; I25.5 Ischaemic cardiomyopathy; I42.0 Dilated cardiomyopathy; I42.9 Cardiomyopathy, unspecified; I50.0 Congestive heart failure; I50.1 Left ventricular failure; I50.9 Heart failure, unspecified. Data were collected between 2016 and 2021 from the patients' electronic medical records. Medications were documented and counted on admission and discharge for all admissions to hospital throughout the study period.

### Polypharmacy classifications

Polypharmacy was determined using the total number of different medications on discharge after the index admission. No patient was discharged with zero medications and only 5% (*n* = 31) with no polypharmacy (one to four medications). Two categories were defined: polypharmacy (five to nine medications) and hyperpolypharmacy (>10 medications). Modelling as a continuous predictor was considered; however, we opted for categorical definitions to align with established polypharmacy thresholds and to maintain consistency with prior literature.

Medication was defined as each drug therapy prescribed with the intention that it would be continued on a repeated basis, that is, monthly/weekly, including those on an as required basis. Each medication count included all *prescribed* regular medication regardless of prescription source (hospital, primary care). Over‐the‐counter medication and current ‘acute course medications’ were not included in the medication count. All medications were coded using the British National Formulary (edition 87).^16^ Medication regimen complexity index (MRCI)^17^ was calculated on each admission and discharge using MS Excel calculator. MRCI is a tool that quantifies medication regimen complexity beyond the number of medications to include weighted scores for types of prescribed dosage forms, dosing frequency, and additional administration directions. The higher the score, the more complex the regimen.^17^

### Heart failure diagnosis

Two sub‐types of heart failure were defined: patients with LVEF ≤ 40% were classed as having HFrEF; and those with LVEF > 40% were classed as having HFpEF. Although the term ‘HF with mid‐range ejection fraction’ (HFmrEF) was introduced in 2014^18^ and into European guidelines in 2016,^19^ NICE guidelines 2018^7^ did not adopt this definition. We therefore included all patients with an LVEF > 40% as a single group. Patients with primary valvular disease as a cause for their heart failure were excluded.

### Comorbidities

All comorbidities documented in the patients' electronic hospital health records were recorded. The total number of comorbidities was determined for each admission to hospital. Some comorbidities were analysed individually, including hypertension, diabetes, chronic kidney disease, chronic obstructive airways disease, and asthma; whereas others were collected as composite variables. For example, ‘Other respiratory’ included: asbestosis, pulmonary fibrosis, obstructive sleep apnoea, pneumoconiosis, and pulmonary hypertension. If a patient had two comorbidities within a single grouping, the comorbidities were counted separately. The Charlson Comorbidity Index (CCI) was also calculated on each admission.^20^ The following cut points were used: 1–2, mild comorbidity; 3–4, moderate comorbidity; and ≥5, severe comorbidity.^21^

### Frailty

Frailty was estimated using the Rockwood clinical frailty scale (CFS)^22^ at the time of admission to hospital. The CFS was taken from clinical documentation on admission; if not documented, we estimated the CFS from the available information. Where insufficient information was available, no score was calculated. Cut points for ease of analysis were created as follows: CFS 1–3, not frail; CFS 4–5, vulnerable/mild frailty; CFS 6–9, moderate‐to‐severe frailty.

### Sample size

The original PULSE dataset sample size was based on estimating the prevalence of polypharmacy in HF two subtype HF populations using methodology by Daniel.^23^ A minimum of 323 per group (HFrEF/HFpEF) was required to estimate the prevalence of polypharmacy with 10% power and a 5% significance level. The sample size was not calculated specifically for mortality analysis.

### Patient and public involvement

‘Pumping Marvellous’ HF patient charity, NIHR RDS NENC PPI consumer panel and BSH patient panel helped with the development of the aims, objectives and outcomes for the POTION Study ‐ POlypharmacy in hearT failure: Investigating the relatIONship between treatment and outcomes. This paper presents data from this study.

### Statistical analysis

Continuous data are described using mean (with standard deviation) or median (with interquartile ranges) depending on distribution or where the median (a whole number) was clinically more accurate. Categorical data are described using number and percentages. Continuous variables were compared using the Student *t*‐test or paired *t*‐test as appropriate, and categorical variables using either chi‐squared test or Fisher's exact test. Where Fisher's exact test was not feasible, chi‐squared testing was used. *t*‐tests and ANOVA were used to compare means, and where data were not normally distributed, Mann–Whitney *U* tests and Kruskal–Wallis tests were used as appropriate. Missing data were excluded from analysis rather than imputed.

Mortality analysis used the number of medications on discharge from the first HF hospitalisation. We used Cox proportional hazard (CPH) models to explore the relation between polypharmacy and mortality. We constructed multivariable models using sex, age, frailty and comorbidity as covariates to account for their known association with polypharmacy and influence on outcome.^24^, ^25^ We used Kaplan–Meier curves to illustrate the results.

We used inverse probability‐weighted regression analysis (IPWRA) to estimate the average treatment effect (ATE). Propensity scores were generated on age, sex, CCI and CFS. Overlap was visually confirmed with stabilised weights (no trimming required). Stabilisation reduces the influences of outlying responses to improve the balance between groups. The weights for each individual are calculated based on the inverse probability of being exposed. By applying the weights, the cohort population is increased, creating a pseudopopulation where confounders are equally distributed between the groups.^26^ The weighted outcome model then produced both the ATE and the potential outcome mean (POmean). IPWRA is doubly robust, meaning estimates remain unbiased if either the propensity score model or the outcome model is correctly specified and therefore protects against confounding and model misspecification beyond that of standard Cox regression.^27^, ^28^

As above, IPWRA gives two outputs:

1. ATE, which is the absolute difference in survival probability between groups, and
2. POmean, which is an estimate of the chance of survival for the whole follow‐up period.

The rationale for reporting both was to provide a triangulated methodology using Cox models to report familiar hazard ratios and IPWRA (which reweights to a pseudo‐population with a stronger balance of covariates) that is more robust to baseline imbalances.

## Results

**Figure 1:**
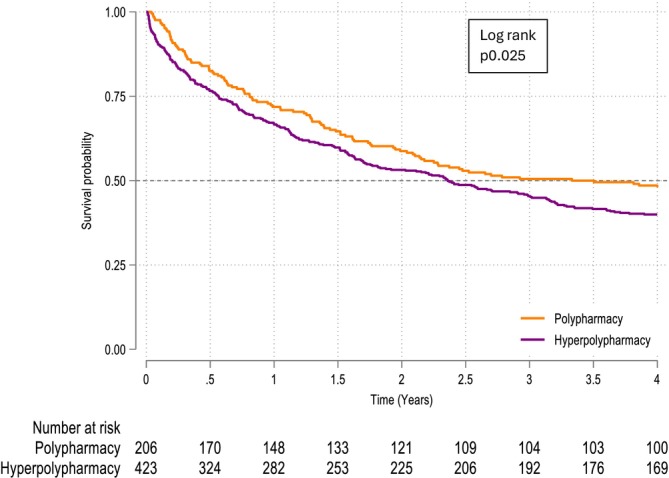
Kaplan–Meier survival curve for polypharmacy from first admission to hospital with heart failure.

A total of 660 people with HF was included in the study covering almost 6 years in total. Median follow‐up to either death or study end was 2.9 years (25th and 75th quartiles 1.6 and 4.5). By the end of the study, 59% of patients had died (*Figure* *1*). Patients with hyperpolypharmacy were older, with a higher ejection fraction (EF) and NT‐proBNP than patients with polypharmacy. Patients with hyperpolypharmacy had more comorbidities, higher CCI, and higher CFS (*Table* *1*). The rate of polypharmacy at discharge was similar in the two HF subgroups, as was MRCI (*Table* *2*). It was rare to be discharged from the hospital with no polypharmacy (5%, *n* = 31). The prescribing rates of individual HF medications did not differ with increased polypharmacy (*Table* *1*). There was no difference in the prescribing of HF medications between the polypharmacy and hyperpolypharmacy groups.

**Table 1:** Patient characteristics of those discharged with polypharmacy versus hyperpolypharmacy (*n* = 593, excludes those who did not survive to discharge and those with no polypharmacy)

	Polypharmacy	Hyperpolypharmacy	*P*
Number patients (%)	206 (34.7)	387 (65.3)	
Age (years), mean (SD)	74.8 ± 13.9	77.0 ± 10.3	0.03
Sex (%)			
Men (%)	120 (58.3)	206 (53.2)	0.242
Female (%)	86 (41.7)	181 (46.8)	
Deprivation index (IMD)			
Median [IQR]	3. [2, 5]	2 [2, 5]	0.09
EF mean (SD)	40.2 ± 16.4	43.0 ± 14.2	0.03
NT‐proBNP [IQR]	4112 [2074, 8540]	5302 [2670, 11 476]	0.007
Length of stay [IQR]	7 [4, 11]	7 [4, 12]	0.21
NYHA class*			
I (%)	3 (1.5)	2 (0.5)	0.55
II (%)	35 (17.2)	60 (15.5)	
III (%)	136 (66.2)	256 (66.3)	
IV (%)	31 (15.2)	68 (17.8)	
Oedema*			
None (%)	37 (18.1)	64 (16.6)	0.27
Mild (%)	40 (19.6)	91 (23.6)	
Moderate (%)	71 (34.8)	149 (38.6)	
Severe (%)	56 (27.5)	82 (21.2)	
Co‐ morbidities [IQR]	5 [3, 6]	7 [5, 8]	<0.001
CCI			
Median [IQR]	5 [4, 7]	6 [5, 7]	<0.001
Clinical frailty scale*			
Median [IQR]	4 [3, 5]	4 [4, 5]	<0.001
HF medication			
Raasi	132 (64.1)	254 (65.6)	0.71
BB	168 (81.6)	332 (85.8)	0.18
MRA	76 (36.9)	126 (32.6)	0.29
SGLT2i	2 (1.00)	9 (2.3)	0.34
Triple therapy	53 (25.7)	87 (22.9)	0.36

* Missing data, percentage based on actual data.

Data are expressed as mean ± SD, median [interquartile range], or number (%). Student t‐test compared means, Mann–Whitney U test compared medians, ANOVA and Kruskal Wallis tests compared categorical data. HFrEF = Heart failure reduced ejection fraction, HFpEF = Heart failure preserved ejection fraction, IMD = *Index of Multiple Deprivation,* NYHA = New York Heart Association classification, EF = Ejection fraction, CCI = Charlson comorbidity index. HF = heart failure, Raasi = Renin angiotensinogen aldosterone system inhibitor (ACEi/ARB/ARNI), BB = Beta‐blocker, MRA = Mineralocorticoid Receptor Antagonist, SGLT2i = sodium‐glucose co‐transporter 2. Triple Therapy = ACEi/ARB/ARNI + BB + MRA.

**Table 2:** Polypharmacy on discharge from first hospital heart failure admission

	HFrEF	HFpEF	*P* value
Total Number of medicines	10 [8, 13]	10 [8, 14]	0.72
None	0	0	0.64
No polypharmacy	18 (5.4)	13 (4.0)	
Polypharmacy	101 (30.5)	105 (31.9)	
Hyperpolypharmacy	212 (64.1)	211 (64.1)	
MRCI	31.2 (14.3)	32.3 (13.8)	0.34

HFrEF = Heart failure reduced ejection fraction, HFpEF = Heart failure preserved ejection fraction, MRCI = Medication regimen complexity index. No medication = 0 medications, No polypharmacy = 1–4 medicines, Polypharmacy = 5–9 medicines, Hyperpolypharmacy ≥10 medicines.

There was no correlation between the *Index of Multiple Deprivation* (IMD) and any of CCI, CFS, or total comorbidity. There was a positive correlation between the number of comorbidities and CCI. CFS correlated with both CCI and the number of comorbidities. The number of medications at discharge correlated weakly with IMD, CCI, CFS, and total comorbidities (*Table* *3*).

**Table 3:** Correlation of variables

		IMD	CCI	Total comorbidities	CFS	Total number medicines at discharge
IMD	**Corr** *P* Obs	**1** ‐ 660				
CCI	**Corr** *P* Obs	**0.04** 0.28 659	**1** ‐ 659			
Total comorbidities	**Corr** *P* Obs	**0.02** 0.67 660	**0.61** * <0.001 659	**1** ‐ 660		
CFS	**Corr** *P* Obs	**−0.02** 0.63 574	**0.40** * <0.001 573	**0.36** * <0.001 574	**1** ‐ 574	
Total number medicines at discharge	**Corr** *P* Obs	**−0.12** * 0.002 626	**0.29** * <0.001 626	**0**.21* <0.001 547	**0**.43* <0.001 626	**1** 626

CCI = Charlson comorbidity index; CFS = Clinical frailty scale; Corr = Correlation coefficient; IMD = Index of Multiple Deprivation,

*P* = *P* value, Obs = Number of observations.

* significant at <0.05.

### CPH models

**Figure 2:**
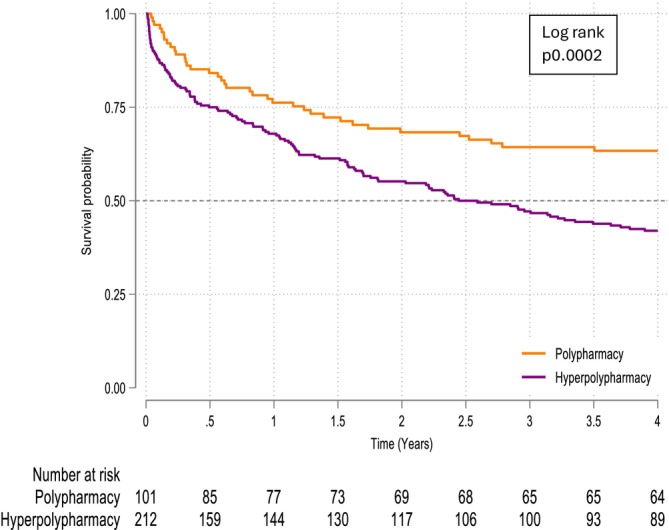
Kaplan–Meier survival curve for polypharmacy for people with HFrEF from first admission to hospital with heart failure.

The univariable model showed that hyperpolypharmacy was associated with a 29% higher mortality than polypharmacy. HFpEF was associated with a 31% higher mortality compared to HFrEF (*Figure* *2*, *Table* *3*). However, after adjusting for age, sex, CCI, CFS, number of drugs, and MRCI, there was no longer a statistically significant association between either polypharmacy or the type of heart failure and outcome. Each additional year of age was associated with a 3% greater mortality; each additional unit increase in CCI was associated with a 12% greater association with mortality; each additional unit increase in CFS was associated with a 28% greater association with mortality; each additional point increase in MRCI was associated with a 4% greater association with mortality; and each additional medication was associated with an 11% lower association with mortality.

In people with HFrEF in the univariable model, hyperpolypharmacy was associated with twice the mortality compared to polypharmacy. However, after adjusting for sex, age, CFS, and CCI, there was no longer a statistically significant association between polypharmacy and outcome. Association with mortality was 2% higher for each additional year of age; 31% higher for each unit increase in CCI; and 40% higher for every one‐point increase in CFS (*Figure* *2*, *Table* *4*).

**Table 4:** Univariable and multivariable analysis of factors related to mortality

	Total cohort (*n* = 629*)						HFrEF (*n* = 253^†^)						HFpEF (*n* = 271^†^)					
	Univariable HR	95% CI	*P*	Multivariable HR	95% CI	*P*	Univariable HR	95% CI	*P*	Multivariable HR	95% CI	*P*	Univariable HR	95% CI	*P*	Multivariable HR	95% CI	*P*
HFpEF	1.31	1.07–1.61	0.009	1.03	0.80–1.30	0.85	‐	‐	‐	‐	‐	‐	‐	‐	‐	‐	‐	‐
Hyper polypharmacy	1.29	1.03–1.61	0.025	0.80	0.56–1.16	0.24	1.95	1.36–2.82	<0.001	0.65	0.37–1.15	0.14	0.93	0.70–1.24	0.63	0.75	0.45–1.23	0.25
Female	0.95	0.77–1.17	0.64	0.69	0.54–0.89	0.003	0.98	0.71–1.34	0.88	0.77	0.53–1.13	0.18	0.86	0.65–1.13	0.27	0.64	0.67–0.88	0.006
Age	1.05	1.04–1.06	<0.001	1.03	1.02–1.05	<0.001	1.06	1.05–1.08	<0.001	1.02	1.00–1.04	0.08	1.04	1.02–1.05	<0.001	1.03	1.01–1.05	0.001
CCI	1.24	1.18–1.30	<0.001	1.12	1.05–1.20	0.001	1.41	1.32–1.51	<0.001	1.31	1.18–1.46	<0.001	1.09	1.02–1.18	0.015	1.00	0.91–1.10	0.95
CFS	1.42	1.31–1.55	<0.001	1.28	1.15–1.42	<0.001	1.65	1.45–1.87	<0.001	1.40	1.20–1.65	<0.001	1.22	1.08–1.39	0.001	1.18	1.03–1.35	0.018
Number of drugs	1.01	0.99–1.04	0.30	0.89	0.80–0.99	0.031	1.04	1.00–1.08	0.07	0.88	0.75–1.03	0.13	1.00	0.96–1.03	0.80	0.94	0.83–1.08	0.39
MRCI	1.01	1.00–1.02	0.04	1.04	1.01–1.06	0.006	1.01	1.00–1.02	0.019	1.04	0.99–1.08	0.13	1.00	0.99–1.01	0.66	1.03	1.00–1.07	0.07

Hyperpolypharmacy = ≥10 medications. CCI = Charlson comorbidity index, CFS = Clinical frailty scale, MRCI = Medication regimen complexity index.

* Exclude those who did not survive to discharge.

^†^ Exclude those who did not survive to discharge, those with no polypharmacy (one to four medications) and with missing variables.

**Figure 3:**
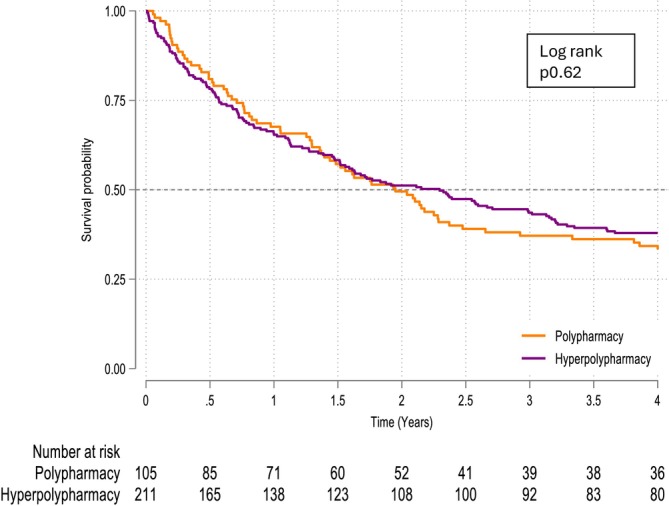
Kaplan–Meier survival curve for polypharmacy for people with HFpEF from first admission to hospital with heart failure.

In people with HFpEF, hyperpolypharmacy was not associated with mortality (*Figure* *3*, *Table* *4*). However, in the adjusted model, female sex was associated with a 36% lower mortality. Mortality was associated with 3% higher for each additional year of age; 18% higher for every one‐point increase in CFS; and 3% higher for each additional point increase in MRCI. CCI was not associated with mortality.

For the adjusted Cox model the contrast was hyperpolypharmacy versus polypharmacy and this was not statistically significant. The HR was <1 or >1; but the CI crossed unity, this indicates imprecision and could infer small harm or small benefit and therefore no defining association in either direction.

### IPWRA (*Table* *5*)

**Table 5:** ATE and POmean of polypharmacy on mortality conditioning on age, sex, CCI and CFS (HFrEF vs. HFpEF)

		Coefficient	95% CI	*P*
Total population				
ATE	Polypharmacy vs. hyperpolypharmacy	−0.10	−0.19 to −0.02	0.02
POmean	Polypharmacy	0.69	0.62–0.77	<0.001
	Hyperpolypharmacy	0.59	0.54–0.64	<0.001
HFrEF				
ATE	Polypharmacy vs. hyperpolypharmacy	−0.26	−0.43 to −0.09	0.003
POmean	Polypharmacy	0.80	0.64–0.95	<0.001
	Hyperpolypharmacy	0.54	0.46–0.61	<0.001
HFpEF				
ATE	Polypharmacy vs. hyperpolypharmacy	−0.04	−0.15 to 0.08	0.55
POmean	Polypharmacy	0.67	0.58–0.76	<0.001
	Hyperpolypharmacy	0.64	0.57–0.71	<0.001

HFrEF = Heart failure reduced ejection fraction, HFpEF = Heart failure preserved ejection fraction, Polypharmacy = 5–9 medications, Hyperpolypharmacy = ≥10 medications.

Negative coefficient = mortality more likely, positive coefficient = mortality less likely.

ATE = Absolute difference in survival probability.

POmean = Probability of survival for each group over the follow‐up period.

The ATE showed that hyperpolypharmacy was associated with a 10% higher risk of death compared to polypharmacy. In patients with HFrEF, the ATE for hyperpolypharmacy was associated with 26% higher mortality than polypharmacy. In patients with HFpEF, hyperpolypharmacy was not associated with mortality. The IPWRA, however, gave a marginal, population‐average ATE and suggested greater mortality risk with hyperpolypharmacy overall (10%) and particularly for HFrEF (26%).

The POmean showed that the chance of survival to the end of follow‐up was 69% for those with polypharmacy and 59% for those with hyperpolypharmacy. In patients with HFrEF, the chance of survival to the end of follow‐up was 80% for those with polypharmacy and 54% for those with hyperpolypharmacy. For patients with HFpEF, the chance of survival to the end of follow‐up was 67% with polypharmacy and 64% with hyperpolypharmacy.

## Discussion

We explored the association between polypharmacy and mortality in people with heart failure. In unadjusted analyses, we found a significant association between greater polypharmacy and higher mortality. Almost all patients leaving the hospital after the first admission with HF are subjected to polypharmacy; only 5% were not. This finding alone has enormous implications for patients, their carers and health care systems. Polypharmacy increases the risk of adverse drug events (side effects, drug interactions and adverse drug reactions), medication errors, non‐adherence, and reduced quality of life.^29^, ^30^ Managing the adverse consequences of polypharmacy further increases healthcare utilisation and costs, beyond that of the cost of the drugs.^31^

Because increasing numbers of medications are associated with both increasing numbers of comorbidities and frailty, we adjusted analyses for age, sex, Charlson comorbidity index, and clinical frailty score. In the adjusted Cox model, we found that polypharmacy was no longer associated with mortality, but the IPWRA model suggested that hyperpolypharmacy was linked with worse survival. The difference likely reflects the way that IPWRA is able to adjust, more robustly, for baseline differences between the polypharmacy and hyperpolypharmacy groups. In the Cox model, the association between frailty, comorbidity, and numbers of medications prescribed may be obscuring the relation between polypharmacy and worse outcome. Both models therefore provide valuable complementary insights: Cox regression suggests that age, sex, CCI, and CFS are strong mortality predictors for HF irrespective of HF subgroup, whilst the IPWRA indicates that polypharmacy may still contribute to mortality risk in HFrEF after adjusting for confounders. Taken together, these analyses point to, at most, a modest independent association in HFrEF and no clear association for HFpEF.

In univariable analysis, patients with HFpEF had a higher association with mortality than patients with HFrEF, even though the numbers of medications and the medicine complexity index were the same in both groups. Those with hyperpolypharmacy also had a higher association with mortality. The effect of adjusting the models for covariates reduced the effect both of HF phenotype and level of polypharmacy on mortality. On splitting the data by HF subtype, univariable analysis showed association with mortality and increased polypharmacy in the HFrEF population that was not present in HFpEF. Age, sex, CCI, and CFS had a greater influence than polypharmacy in multivariable analysis. Whilst the complex relations between age, comorbidity, and frailty make it difficult to detect any independent effect of polypharmacy, the IPWRA suggests that there is a residual association between polypharmacy and worse mortality beyond its correlation with disease burden. Although hyperpolypharmacy often reflects greater frailty and multimorbidity, it might thus also carry an additional risk particularly in HFrEF. Our findings are consistent with prior observational studies showing an association between polypharmacy and adverse outcomes in heart failure.^3^, ^32^, ^33^, ^34^

Polypharmacy is common in people with heart failure^1^ and in advancing age^35^ due to multiple comorbidities.^36^ National and international treatment guidelines often mandate complex treatment regimens, which can make medical life for an individual patient with, say, diabetes, left ventricular systolic dysfunction and COPD, extremely burdensome. Those with HFrEF will likely accumulate a polypharmacy medication regimen treating HF alone with GDMT, whereas GDMT for HFpEF will not impose the same level of polypharmacy. The underlying pathophysiological differences could influence both the degree of polypharmacy and the contributory drug classes. HFrEF is characteristically a reduced contractility problem most commonly caused by IHD,^37^ whereas HFpEF, due to ventricular stiffness and impaired relaxation, is influenced by systemic comorbidities^38^, ^39^ rather than direct myocardial insult. The high level of polypharmacy in HFpEF could be assumed to be due to the higher burden of comorbidities, known to be associated with higher risk of mortality, more so than HFrEF.^40^ This may begin to explain the difference in association with mortality between the two HF subtypes.

Polypharmacy can be seen as both a risk marker and a modifiable risk factor. It signals underlying multimorbidity or disease burden and its associations with adverse outcomes such as falls, adverse drug events, non‐adherence, and hospital admission. However, medication burden is potentially modifiable with improvements in disease control or intentional interventions by healthcare professionals. In particular, the risk of adverse outcomes can be reduced with interventions, such as ‘deprescribing’ to discontinue inappropriate medications. Thus, the level of polypharmacy can be modified to lower risk.

Polypharmacy is often described as ‘appropriate’ and ‘inappropriate’. No universal definition of appropriate or inappropriate polypharmacy exists. Appropriate polypharmacy has been described as ‘the use of multiple medications that are clinically indicated, optimised and prescribed according to best evidence. They extend life expectancy and improve quality of life for the patient’.^30^ Appropriate polypharmacy occurs when multiple medications are prescribed in a manner that is clinically justified, taking into account guidelines and applying to the individual patient whilst considering multiple comorbidities, therapeutic necessity, risk–benefit and patient goals.

Appropriate polypharmacy should follow a patient‐centred approach to treatment decisions with appropriate monitoring and adjustment to ensure ongoing benefit. Inappropriate polypharmacy arises when medications are prescribed unnecessarily, without clear benefits, or when the risks outweigh the benefits. This can lead to adverse outcomes such as side effects, drug interactions, overprescribing, and increased healthcare costs.^41^ Thus, for example, whilst an HbA1c above some arbitrary number might mandate increased anti‐diabetic therapy in a guideline, it may be entirely inappropriate in a very frail patient with multiple other conditions who is already taking 10 medications. Due to the difference in GDMT, appropriate and inappropriate polypharmacy may well differ depending on a HFrEF or HFpEF diagnosis. Greater understanding of appropriate and inappropriate polypharmacy in HF is crucial and should be included in future studies. However, this study shows that the absolute number of medications still matters in terms of patient outcomes. In clinical practice, the number of medicines is important in clinical risk flagging to identify those who require medication reviews. Those with a higher medication count are associated with greater potential for inappropriate medication.^42^

Generally, the higher the number of individual comorbidities, the higher the number of medications prescribed.^36^ The number of comorbidities a patient has diagnosed is rarely adjustable, but the number of medications to treat each one potentially is. De‐prescribing is the systematic process of discontinuing medications that are no longer needed, potentially harmful, or not providing an intended benefit.^43^

Although de‐prescribing often features in clinical practice guidelines, some guidelines do not contain clear, actionable recommendations on *how* to de‐prescribe.^44^ Barriers to de‐prescribing include: lack of knowledge and training; lack of time; resistance from patient; fear of consequences; and communication breakdown.^45^ Many comorbidities potentially have overlapping treatment options and maximising the overlap may help reduce polypharmacy burden. What remains unclear is whether all polypharmacy confers the same risk. Or is the degree of risk influenced by appropriate versus inappropriate medication.

Many patients admitted to hospital for heart failure are not prescribed life‐prolonging medication for which they would be eligible and are often prescribed medication known not to improve outcomes (and which might cause harm).^34^ The average length of hospital stay for a HF admission in the United Kingdom is 8 days.^46^ This gives multiple opportunities to: review medication; ensure medications are taken as prescribed; discuss the aims of treatment; and appraise understanding. During such time, utilising the vital skills of a pharmacist to support in making rational decisions about what medications to *stop* as well as what medications to start could be beneficial. Future research should explore the extent to which appropriate versus inappropriate polypharmacy influences patient outcomes.

Frailty is strongly associated with higher mortality, particularly in patients with heart failure and polypharmacy.^47^, ^48^, ^49^ Assessing and addressing frailty, alongside medication reviews and addressing polypharmacy, should be part of standard care. Guidelines^7^, ^8^, ^9^ recommend multidisciplinary team (MDT) care for patients with HF, underscoring the importance of this approach in standard practice. Multidisciplinary heart failure teams (which should include cardiologists, nurses, pharmacists, dietitians and geriatricians) can reduce hospitalisation and healthcare costs,^50^ improve medication adherence, and improve outcomes.^51^ A hospital admission is the ideal opportunity to utilise the diverse skills of an MDT to provide holistic comprehensive care that addresses the multifaceted needs of HF patients, including medication review, optimisation, and deprescribing.

It is recognised that this paper presents polypharmacy data from the first heart failure hospitalisation. Many patients will have had an HF disease trajectory prior to this point, of varying lengths, that has influenced the number of medications prescribed. Mortality post HF hospitalisations is high and a marker of poor prognosis,^52^ potentially influencing the outcome of this study. Understanding polypharmacy in patients with HF from diagnosis could provide earlier opportunities for interventions to address polypharmacy and related adverse events. Clinically, these results highlight the need for regular medication review, especially in patients with HFrEF, with a focus on both the necessity and appropriateness of prescribed therapies. The study's observational nature and residual confounding limit the ability to infer causality.

## Conclusions

Polypharmacy is associated with worse mortality following hospitalisation for heart failure, particularly in those with HFrEF. However, some of the association may be driven by the relation between polypharmacy, age, comorbidities, and frailty. Future research should focus on prescribing strategies for HFrEF to prioritise reducing medication burden where clinically appropriate to mitigate potential risks associated with polypharmacy.

### Limitations

Sample size was not calculated for mortality analysis and therefore may introduce error and reduce generalisability. We only assessed polypharmacy at the fixed time point of first admission to hospital with heart failure. We cannot know what happened to prescribing after discharge, which may weaken any association between polypharmacy and outcome. Study data is from a single institution with a particular demographic (relatively deprived, post‐industrial, mainly white) and so might not be representative of a wider population.

## Funding

Development of the PULSE database is part of a wider study, the POTION study (POlypharmacy in hearT failure: Investigating the relatIONship between treatment and outcomes) that is supported by a research fellowship from the British Society for Heart Failure and a training grant from Pharmacy Research UK.

## Conflict of interest

None declared.

## Disclosure

Janine Beezer declares that she has no conflict of interest. Andrew Clark declares that he has no conflict of interest. Adam Todd declares that he has no conflict of interest. Andrew Kingston declares that he has no conflict of interest. Andrew Husband declares that he has no conflict of interest.

## Data Availability

The datasets generated during and/or analysed during the current study are available from the corresponding author on reasonable request.
